# Discrimination and school belonging independently but not interactively associated with suicidal thoughts among adolescents: a brief report

**DOI:** 10.3389/frcha.2026.1855058

**Published:** 2026-10-30

**Authors:** Jocelyn Meza, Elena Maker Castro, Juliane L. Martinez, Grace M. Hindmarch, Sunhye Bai

**Affiliations:** 1 Department of Psychiatry & Biobehavioral Sciences, University of California Los Angeles, Los Angeles, CA, United States; 2 Department of Psychology, Bates College, Lewiston, ME, United States; 3 Department of Health Policy & Management, University of California Los Angeles, Los Angeles, CA, United States; 4 The Ballmer Institute for Children’s Behavioral Health, University of Oregon, Portland, OR, United States

**Keywords:** adolescent, civic engagement, discrimination, school belonging, suicidal ideation

## Abstract

**Introduction:**

Discrimination has been identified as a significant risk factor for suicidal ideation (SI) in adolescence, yet few studies have examined protective factors that may interact with this association.

**Methods:**

Using cross-sectional data from the 2021 California Health Interview Survey (CHIS), this brief report examines whether racial/ethnic discrimination is associated with higher odds of SI, and whether civic engagement and sense of belonging moderate this concurrent association. The sample included 1,169 adolescents aged 12–17 years (*M* = 14.57; *SD* = 1.67).

**Results:**

Binary logistic regressions revealed that racial/ethnic discrimination was significantly associated with higher odds of SI (*aOR* = 2.29, 95% CI [1.46, 3.58], *p* < .001), while sense of belonging at school was significantly associated with lower odds of SI (*aOR* = 0.66, 95% CI [0.55, 0.78], *p* < .001). Contrary to hypotheses, civic efficacy (*aOR* = 0.96, 95% CI [0.87, 1.06], *p* = .389), community involvement (*aOR* = 0.83, 95% CI [0.53, 1.32], *p* = .473) and community leadership (*aOR* = 1.29, 95% CI [0.79, 2.09], *p* = 0.448) were not significantly associated with reduced odds of SI. None of the interactions were significant (*p* > .05), though moderation analyses may have been underpowered.

**Discussion:**

These findings emphasize that discrimination and school belonging were independently linked to SI, but no interactive effects emerged. Given that school belonging was linked to lower odds of SI, implementing school-based strategies that foster belonging may strengthen adolescent suicide prevention efforts in the U.S.

## Introduction

Suicide is the second leading cause of death for adolescents in the United States, with rates increasing 36% between 2000 and 2021 (1). According to the Structural Racism and Suicide Prevention Systems framework, structural racism and interpersonal discrimination operate across multiple systems, including schools, communities, and institutions, which are crucial contexts influencing suicide among adolescents (2), while also identifying opportunities where protective resources can mitigate risk. This framework informs the hypothesis that structural and interpersonal discrimination would be positively associated with SI, and that civic engagement and school belonging—as resources embedded within community and school systems—could buffer this risk. Simultaneously, civic engagement, often defined by an individual's participation in volunteer and political activities that contribute to their community and societal well-being, may buffer against these risks by fostering a school belonging and purpose (3, 4). However, very few studies have examined the effect of civic engagement on adolescents and how it may interact with experiences of discrimination in the context of suicide. Therefore, this brief report will examine (1) the magnitude of the association between racial/ethnic discrimination and SI among adolescents, and (2) whether civic engagement (i.e., community leadership, community involvement, and civic efficacy) and school belonging moderates this association.

### Discrimination as a risk factor for suicidal ideation

Existing literature highlights discrimination as an immense stressor undermining mental health (5, 6), which is consistent with the Stress Process Theory. This theory highlights discrimination as a chronic stressor with significant effects on well-being (7). Adolescents who experience discrimination report elevated levels of loneliness, depression, psychological distress, and anxiety—precursors to suicide—and discrimination has also been significantly associated with SI (8–11). A study among adolescents found that racial discrimination was associated with serious psychological distress within the past 30 days, past-year SI, and past-year suicide attempt (SA) (8). Another study by Oh et al. (12) found that the highest level of “everyday discrimination” reported among young adults was associated with 5.6 times greater odds of SI, 4.8 times greater odds of suicide plan, and 5.3 times greater odds of planned SA. Adolescents who have experienced discrimination may perceive their environment as hostile and unchangeable, increasing risk for SI, and highlighting the need to identify factors that reduce this harm (13, 14).

### School belonging as a protective factor

School belonging, particularly in adolescence, can function as a psychosocial resource that can buffer the effects of stress exposure (7), and is associated with positive mental health outcomes, such as enhanced psychological well-being and reduced distress (15, 16). In the context of discrimination, a robust sense of belonging can critically mitigate the risk of negative mental health outcomes, such as depression and anxiety (17, 18). For instance, studies have shown that higher levels of school belonging correlate with reduced depressive symptoms, increased social acceptance, and higher sense of self-worth among college students (19–21). Furthermore, research has demonstrated that a higher sense of belonging within the school context is associated with students' confidence in their ability to persist through graduation, even when accounting for experiences of discrimination (22). While most of this work has focused on college students, limited research has explored these associations among adolescent samples, and few studies have specifically examined the impact of belonging at school.

### Civic engagement as a protective factor

Civic engagement is a broad, multidimensional term that encompasses political and prosocial participation in society and includes behavioral (e.g., participation in organizations, leadership in the community) and psychological dimensions (e.g., sense of efficacy) (23, 24). Adolescents who are civically engaged tend to have a stronger purpose in life (24–26), supporting well-being within and beyond adolescence (3, 23, 27, 28). Importantly, civic engagement can serve as a form of coping, which is aligned with the Stress Process Theory's framework of psychosocial resources as mechanisms that can help mitigate the adverse mental health outcomes associated with discrimination (7, 28, 29). Experiences of discrimination can alienate racially/ethnically minoritized adolescents from civic life (29), and it can also catalyze action to create social change (30). Notably, there may be important intersections between belonging and civic engagement, such that civic organizational spaces may foster a stronger sense of inclusion, support, and validation among marginalized youth (31). As the literature on adolescent civic engagement and well-being continues to evolve, more attention is merited on more severe forms of mental health challenges, including SI (32).

### Current study

Overall, more studies are needed to examine the relationship between racial/ethnic discrimination and SI among adolescents, and to examine key protective social factors that can mitigate this association. While a recent study found that school connectedness, another measure of school belonging, partially buffered the effect of bullying victimization (main stressor) on suicide risk among U.S. high school adolescents (33), the current study instead focuses on racial/ethnic discrimination as the main stressor, assessing civic engagement and school belonging as simultaneous protective moderators. Using statewide representative data, our study advances the field by examining whether civic engagement and school belonging can buffer the mental health risks specifically posed by racial/ethnic discrimination, a stressor distinct from peer-based bully victimization. Guided by the Structural Racism and Suicide Prevention Systems framework and the Stress Process Theory (7), we hypothesized that discrimination would be associated with higher odds of SI and that civic engagement and school belonging—both psychosocial resources—would moderate the association between discrimination and SI.

## Methods

### Participants

Data was analyzed from the 2021 wave of the California Health Interview Survey (CHIS), a cross-sectional, multimodal (web and phone), statewide survey. A sample of 1,169 adolescents (*M* = 14.57; *SD* = 1.67) participated in the 2021 CHIS survey. In a household, CHIS collects socio-demographic and health-related information from one adult respondent who was randomly selected to complete the interview. Consent was obtained from the adolescent's parent or guardian to interview one randomly selected youth in the household between the ages 12 and 17 years. The sample consisted of 52.3% males (*N* = 611; weighted: 47.0%) and 47.7% females (*N* = 558; weighted: 53.0%), 36.9% (weighted: 51.5%) of participants were Hispanic, 38.6% (weighted: 27.2%) were non-Hispanic White, 1.9% (weighted: 4.6%) were African American, 14.4% (weighted: 10.6%) were Asian, 0.3% (weighted: 0.6%) were Native Hawaiian/Pacific Islander, and 7.9% (weighted: 5.5%) were 2 or more races.

### Measures

#### Racial/ethnic discrimination

Racial/ethnic discrimination was measured by asking the following question: “*Have you ever been treated or judged unfairly because of your race or ethnic group*?” Answer choices for this question were recoded to 0 = “No” and 1 = “Yes.”

#### Civic engagement

Four questions assessed three aspects of civic engagement: (1) community leadership (2) community involvement, and (3) civic efficacy. To measure community leadership the following question was asked: “*In the past 12 months, have you volunteered to organize or lead efforts to help solve problems in your community?”* The following question measured community involvement: “*In the past 12 months, did you participate in any clubs or organizations outside of school, other than sports, like YMCA or Boys and Girls Club?”* Answer choices for the questions measuring community leadership and community involvement were recoded to 0 = “No” and 1 = “Yes”. To measure civic efficacy, the following two Likert-scale questions were asked: “*Do you think you could express your views in front of a group of people?”* and “*Do you think you could contact an elected official or someone else in government who represents your community?”* Response options were: 1 = “Definitely could not,” 2 = “Probably could not,” 3 = “Maybe could,” 4 = “Probably could,” and 5 = “Definitely could.” The scores of both questions were averaged, with higher scores indicating higher civic efficacy (*α* = 0.663).

#### School belonging

To measure sense of belonging at school, participants were asked: *How often have you felt a sense of belonging at school?* Participants had the following answer choices: 1 = “All of the time”, 2 = “Most of the time”, 3 = “Some of the time”, 4 = “A little of the time”, and 5 = “Never”. Answers were reverse scored so that higher scores indicated a higher sense of belonging in school settings.

#### Lifetime suicidal ideation

The following question was administered to measure lifetime SI: “*Have you seriously thought about committing suicide?”* Answer choices for this question were recoded to 0 = “No” and 1 = “Yes.”

### Data analytic plan

We conducted logistic regressions to estimate binary SI as the outcome, over and above the control of key covariates: depression symptoms, parent-reported household income, youth race/ethnicity (i.e., Hispanic, Black, Asian, Native American with non-Hispanic White youth as reference), age, and gender. Demographic characteristics are reported as unweighted and weighted proportions. All prevalence estimates, correlations, and regression analyses were weighted using CHIS survey weights to produce estimates representative of the broader population of California adolescents. Depression was included as a covariate to estimate the association between discrimination and SI, consistent with prior research examining discrimination and suicide-related outcomes (12, 34, 35). Bivariate associations among categorical (binary) study variables (i.e., discrimination, community involvement, community leadership, and suicidal ideation) were assessed using weighted chi-square tests. Associations between binary and continuous/ordinal variables are reported as point-biserial correlations, which are mathematically equivalent to Pearson correlations for dichotomous-continuous pairs. Associations among ordinal and continuous variables (school belonging, depression, civic efficacy) are reported as Pearson correlations, as no validated procedure currently exists under the applied survey weighting design. All regression analyses were estimated with pseudo-likelihood methods that apply CHIS survey weights resulting in estimates that are representative of the 2021 California population of adolescents. Estimates of uncertainty are computed using replicate weights, resulting in design-based standard errors that reflect the CHIS sampling design. The analyses were done in R using the **survey** package (36). Model fit for multivariable logistic regression was assessed using a design-based Wald chi-square test comparing the full model to a null model with Cox-Snell and Nagelkerke pseudo-*R*^2^ estimates to characterize explanatory power.

Missing data on study variables were minimal and were addressed by CHIS prior to data release using hot desk imputation, with corresponding imputation flags provided for each variable. The percentage of imputed cases ranged from 0% (discrimination) to 1.97% (suicidal ideation); school belonging (1.28%), civic efficacy items (0.60%–0.68%), community involvement (0.26%), community leadership (0.68%), depression (0.68%), and race/ethnicity (0.94%).

## Results

A total of 19.1% (*N* = 223) participants reported lifetime SI. In addition, 16.2% reported experiencing racial/ethnic discrimination. Descriptive statistics for study variables are present in Table 1. The civic efficacy composite score had a weighted mean of 5.90 (*SD* = 1.99, range = 2–10). For the two ordinal variables, weighted response distributions were as follows: school belonging—6.1% never, 12.6% a little of the time, 24.9% some of the time, 31.7% most of the time, and 24.6% all of the time (mode = 4, “Most of the time”); depression—62% all of the time, 18.9% most of the time, 12.0% some of the time, 4.9% a little of the time, and 2.2% none of the time, across response categories 1 through 5, respectively (mode = 1, “All of the time”).

**Table 1:** Correlation among variables of interest.

Measured variables	1	2	3	4	5	6	7
1. Discrimination	–	–	–	–	–	–	–
2. School Belonging	−0.13*	–	–	–	–	–	–
3. Civic Efficacy	−0.02	0.27*	–	–	–	–	–
4. Community Involvement	−0.03	0.13*	0.27*	–	–	–	–
5. Community Leadership	0.10*	0.08*	0.24*	0.30*	–	–	–
6. Suicidal Ideation	0.21*	−0.32*	−0.08*	−0.01	0.07*	–	–
7. Depression	0.18*	−0.38*	−0.16*	−0.01	0.00	0.47*	–
*n* (sample size)	1,169	1,169	1,169	1,169	1,169	1,169	1,169
Range	0 to 1	1 to 5	2 to 10	0 to 1	0 to 1	0 to 1	1 to 5
M (SD)	0.18 (0.38)	3.56 (1.16)	5.90 (1.99)	0.21 (0.40)	0.19 (0.39)	0.18 (0.39)	1.66 (1.01)

* *p* *<* *.05.*

Civic Efficacy is a composite score of two items (range 2–10). School belonging and Depression are single-item ordinal variables (range 1–5); weighted response distributions for ordinal variable: School belonging—Never: 6.1%, A little of the time: 12.6%, Some of the time: 24.9%, Most of the time: 31.7%, All of the time: 24.6% (mode = 4, “Most of the time”); Depression—All of the time: 62.0%, Most of the time: 18.9%, Some of the time: 12.0%, A little of the time: 4.9%, None of the time: 2.2% (mode = 1, “All of the time”). Percentages reflect CHIS-weighted estimates. Weighted chi-square tests (df = 1, *N* = 1,169) were used to assess bivariate associations among categorical (binary) study variables and were consistent in direction and significance with the correlations above: Discrimination × Community Involvement, *χ*^2^ = 0.68, *p* = .414; Discrimination × Community Leadership, *χ*^2^ = 12.87, *p* < .001; Discrimination × Suicidal Ideation, *χ*^2^ = 36.43, *p* < .001; Community Involvement × Community Leadership, *χ*^2^ = 91.07, *p* < .001; Community Involvement × Suicidal Ideation, *χ*^2^ = 0.10, *p* = .748; Community Leadership × Suicidal Ideation, *χ*^2^ = 4.58, *p* = .035.

Correlations (see Table 1) revealed that discrimination was significantly and positively correlated with community leadership (*r* = 0.10, *p* < .01), lifetime SI (*r* = 0.21, *p* < .01), and depression (*r* = 0.18, *p* < .01). Sense of belonging within the school context was significantly and positively correlated with civic efficacy (*r* = .27, *p* < .01), community involvement (*r* = 0.13, *p* < .01), and community leadership (*r* = .08, *p* < .01). However, school belonging was negatively correlated with racial/ethnic discrimination (*r* = −0.13, *p* < .01), lifetime SI (*r* = −0.32, *p* < .01), and depression (*r* = −0.38, *p* < .01). Weighted chi-square tests (*df* = 1, *N* = 1,169) were used to assess bivariate associations among categorical (binary) study variables and were consistent in direction and significance with the correlations above: Discrimination × Community Involvement, *χ*^2^ = 0.68, *p* = .414; Discrimination × Community Leadership, *χ*^2^ = 12.87, *p* < .001; Discrimination × Suicidal Ideation, *χ*^2^ = 36.43, *p* < .001; Community Involvement × Community Leadership, *χ*^2^ = 91.07, *p* < .001; Community Involvement × Suicidal Ideation, *χ*^2^ = 0.10, *p* = .748; Community Leadershi*p* × Suicidal Ideation, *χ*^2^ = 4.58, *p* = .035.

While controlling for depression, household income, race/ethnicity, age and sex, binary logistic regressions revealed that racial/ethnic discrimination significantly associated with higher odds of SI (*aOR* = 2.29, 95% CI [1.46, 3.58], *p* < .001; see Table 2). Contrary to hypotheses, civic efficacy (*aOR* = 0.96, 95% CI [0.87, 1.06], *p* = 0.389), community involvement (*aOR* = 0.83, 95% CI [0.53, 1.32], *p* = .473) and community leadership (*aOR* = 1.29, 95% CI [0.79, 2.09], *p* = 0.448) were not significantly associated with reduced odds of SI. However, sense of belonging at school (*aOR* = 0.66, 95% CI [0.55, 0.78], *p* < .001) was significantly associated with lower odds of SI. Four different interaction terms were added to the main effects models, separately for each moderator, to examine whether civic efficacy, community involvement, community leadership, and school belonging moderate the associations between racial/ethnic discrimination and lifetime SI. All models controlled for main effects, and covariates of depression, household income, race/ethnicity, age, and gender. However, interactions between racial/ethnic discrimination and civic efficacy (Wald *χ*^2^ (1) = 3.90, *p* = .53), community involvement (Wald *χ*^2^ (1) = 0.32, *p* = .57), community leadership (Wald *χ*^2^ (1) = 2.64, *p* = 0.11) and school belongingness (Wald *χ*^2^ (1) = 2.63, *p* = .14) did not improve model fit relative to the main effects model and were not statistically significant. The overall multivariable model significantly improved model fit relative to a null model, Wald *χ*^2^ (10, *N* = 1,169) = 216.48, *p* < .001, and explains a moderate proportion of the variance in lifetime SI (Cox-Snell *R*^2^ = .23; Nagelkerke *R*^2^ = .36).

**Table 2:** Binary logistic regression models of variables associated with lifetime suicide ideation.

Predictor	aOR	95% CI	*p*
Main Effects Models			
Racial/Ethnic Discrimination	2.29	[1.46, 3.58]	<.001
Civic Efficacy	0.96	[0.87, 1.06]	.389
Community Involvement	0.83	[0.53, 1.32]	.473
Community Leadership	1.29	[0.79, 2.09]	.448
School Belonging	0.66	[0.55, 0.78]	<.001
Interaction Models			
Discrimination × Civic Efficacy	0.78	[0.60, 1.02]	.053
Discrimination × Community Involvement	0.71	[0.28, 1.79]	.571
Discrimination × Community Leadership	0.45	[0.16, 1.28]	.109
Discrimination × School Belonging	1.35	[0.91, 2.01]	.137

aOR, adjusted odds ratio; CI, confidence interval. Binary logistic regression no/yes SI as outcome. All models adjust for depression, household income, teen's race/ethnicity, teen's age, teen's gender. The overall multi-variable model significantly improved it relative to a null model, Wald *χ*^2^ (10, *N* = 1,169) = 216.48, *p* < .001, and explains a moderate proportion of lifetime SI (Cox & Snell R^2^ = .23; Nagelkerke R^2^ = .36).

## Discussion

As hypothesized, our results underscore the role of racial/ethnic discrimination as a risk factor associated with higher odds of SI among adolescents. About 20% of participants reported lifetime SI, and 16.2% reported experiencing racial/ethnic discrimination in their lifetime. Our findings also highlighted that even after considering factors such as depression, family income, race/ethnicity, age, and sex, adolescents who experienced racial/ethnic discrimination had more than twice the odds of report SI compared to those who did not experience discrimination.

Our estimates of experiencing discrimination are in line with previous national estimates of high school students across the U.S (37, 38). Recent studies link discrimination as a risk factor among college-age students/young adults, yet few studies have examined this association among adolescents, and have also neglected to examine which modifiable factors can mitigate the risk (13, 39). We hypothesized that civic engagement would reduce the association between discrimination and SI, yet our results did not demonstrate this proposed protective effect, though this null finding should be cautiously interpreted given the limited statistical power to detect interaction effects. Our results may reflect an overall net neutral effect of civic engagement on well-being, such that at times, civic engagement may serve as a coping mechanism, but when enacted within contexts of discrimination, it may not have a protective effect (28, 31). Although sense of belonging in the school context did not moderate the association between discrimination and SI, school belonging was independently associated with reduced odds of SI. Given that other research identifies civic engagement as a pivotal space for fostering belonging (32), there may be more complex indirect pathways between discrimination, civic engagement, school belonging and SI. Indeed, our findings uncovered that civic engagement was positively and significantly associated with a sense of belonging in the school setting. From the lens of the Stress Process Theory (7), these results suggest that not all hypothesized psychosocial resources function as effective buffers against a structurally embedded stressor such as racial/ethnic discrimination.

Our study should be interpreted with limitations in mind. First, our study included cross-sectional data which precluded our ability to examine causality or longitudinal patterns of discrimination and other plausible mediators (i.e., depression). Second, the CHIS data are only generalizable among adolescents in California. Adolescents may experience racial/ethnic discrimination differently in other parts of the country. Third, both the racial/ethnic discrimination and SI variables were assessed using lifetime frames, which impact the ability to determine the proximal relationship between these two variables. Similarly, civic engagement variables did not reflect all aspects and characterization of the multidimensional construct (e.g., civic knowledge, the full range of civic activities, frequency of engagement in activities) and we did not use a full or validated measure to examine this construct, a key limitation when working with large epidemiological samples. Furthermore, we could not examine frequency of experiencing racial/ethnic discrimination, which could have a differential effect on our outcome of interest. In addition, small cell sizes for Native Hawaiian/Pacific Islander (*N* = 4) and African American (*N* = 22) adolescents limit the generalizability of race/ethnicity-specific estimates in our models. These estimates should be interpreted with caution given their limited statistical power.

Equally important to note, our model analyses may have been underpowered to detect true interaction effects. Only 16.2% of the sample reported experiencing racial/ethnic discrimination, and interaction effects typically require larger samples to achieve statistical power. Therefore, our null findings for civic engagement and school belonging as moderators should not be interpreted as evidence of no true effect, but instead as inconclusive given the limited power available. Future research with larger samples of adolescents who have experienced discrimination is needed to evaluate these moderation hypotheses. Furthermore, the discrimination measure captures experiences of unfair treatment due to race/ethnicity across any context, while the belonging measure was specific to the school setting, limiting conclusions about belonging in other settings and its relation to civic engagement in a community setting. This context mismatch between discrimination and belonging measures may have limited our ability to detect a moderating effect, as the stressor and protective resource were not assessed within the same context; school belonging may be more proximal to adolescents' daily experiences and may not buffer discrimination that is experienced through community settings. Future research using context-specific measures of both discrimination and belonging would allow for a more precise test of the moderation hypothesis and should assess belonging across multiple contexts (e.g., school, community). Additionally, all study variables in the analyses were self-reported by adolescents in a survey. The associations among these variables may reflect shared method variance attributable to the single informant and assessment method instead of substantive relationships among the constructs themselves. Future studies with multiple informants (e.g., parents and teachers) and assessment modalities (e.g., administrative records and observations) would help address the extent to which these associations reflect true relationships vs. shared-method variance. In addition to this limitation, the internal consistency for the civic efficacy measure was modest (Cronbach's *α* = .66). Although this value falls slightly below the. 70 threshold, the measure contained only two items, and the Cronbach's alpha is sensitive to scale length (40). Therefore, the observed reliability was considered reasonable given the short length of the measure, although results involving this construct should be interpreted with caution.

### Implications for research and practice

These findings underscore racial/ethnic discrimination as a salient and independent correlate of SI among adolescents. Although civic engagement did not buffer the association between discrimination and SI, our results suggest that school belonging was independently associated with reduced odds of SI. These findings highlight the importance of situating adolescent suicide risk within broader socio-ecological contexts, where discrimination may increase vulnerability, while interpersonal and structural factors (e.g., belonging within schools) may serve as critical points of intervention. Consistent with the Structural Racism and Suicide Prevention Systems framework and Stress Process Theory, these findings suggest that suicide prevention efforts should address structural and interpersonal discrimination and the psychosocial resources that may be linked to reduced risk. Prevention scientists and school-based practitioners may consider integrating belonging-focused strategies, such as culturally responsive classroom programs and staff training on inclusive engagement, into a system of support that can be scalable for reducing and preventing suicide risk, particularly among students who experience racial/ethnic discrimination.

## Data Availability

The data analyzed in this study is subject to the following licenses/restrictions: The data that support the findings of this study are available from the UCLA Center for Health Policy Research's California Health Information Study. Requests to access these datasets should be directed to https://healthpolicy.ucla.edu/.
